# Update on the Roles of Rice MAPK Cascades

**DOI:** 10.3390/ijms22041679

**Published:** 2021-02-07

**Authors:** Jie Chen, Lihan Wang, Meng Yuan

**Affiliations:** National Key Laboratory of Crop Genetic Improvement, National Center of Plant Gene Research (Wuhan), Huazhong Agricultural University, Wuhan 430070, China; shijiechen@webmail.hzau.edu.cn (J.C.); lhwang@webmail.hzau.edu.cn (L.W.)

**Keywords:** MAPK cascade, phosphorylation, rice, growth and development, biotic and abiotic stress, phytohormone signal transduction

## Abstract

The mitogen-activated protein kinase (MAPK) cascades have been validated playing critical roles in diverse aspects of plant biology, from growth and developmental regulation, biotic and abiotic stress responses, to phytohormone signal transduction or responses. A classical MAPK cascade consists of a MAPK kinase kinase (MAPKKK), a MAPK kinase (MAPKK), and a MAPK. From the 75 MAPKKKs, eight MAPKKs, and 15 MAPKs of rice, a number of them have been functionally deciphered. Here, we update recent advances in knowledge of the roles of rice MAPK cascades, including their components and complicated action modes, their diversified functions controlling rice growth and developmental responses, coordinating resistance to biotic and abiotic stress, and conducting phytohormone signal transduction. Moreover, we summarize several complete MAPK cascades that harbor OsMAPKKK-OsMAPKK-OsMAPK, their interaction with different upstream components and their phosphorylation of diverse downstream substrates to fulfill their multiple roles. Furthermore, we state a comparison of networks of rice MAPK cascades from signal transduction crosstalk to the precise selection of downstream substrates. Additionally, we discuss putative concerns for elucidating the underlying molecular mechanisms and molecular functions of rice MAPK cascades in the future.

## 1. Introduction

The mitogen-activated protein kinase (MAPK) cascades have been designated to be highly conserved signal transduction modules in eukaryotes with diverse functions by linking different extracellular stimuli to a wide range of intracellular responses [1,2]. A complete MAPK cascade mainly consists of three kinases, including a MAPK kinase kinase (MAPKKK or MEKK), a MAPK kinase (MAPKK or MEK), and a MAPK (MPK). Upon sensing external stimulus signal, MAPKKKs phosphorylate and activate MAPKKs, the activated MAPKKs subsequently phosphorylate MAPKs, and finally the activated MAPKs phosphorylate a large number of specific downstream substrates, such as transcription factors, chromatin remodeling factors, kinases or other enzymes, leading to reprogramming of transcriptome and proteome in the whole cell. The sequential phosphorylation is fundamental for MAPK cascade-mediated signal transduction and interactions between MAPK proteins and their substrates [3].

In plants, the MAPK cascades play essential roles in growth and developmental regulation, biotic and abiotic stress responses, phytohormone signal transduction or responses [1,4,5,6,7]. After receiving external signals, plant MAPKKKs mostly phosphorylate the two conserved serine (S) and threonine (T) residues in the S/T-X5-S/T (X is any amino acid) motif of MAPKKs and activate MAPKKs. The activated MAPKKs in turn phosphorylate both the threonine (T) and the tyrosine (Y) in the T-D-Y or T-E-Y motif of MAPKs and activate MAPKs. However, plant MAPK cascade-mediated signal transduction needs to be precisely regulated, as continuous activation or suppression of MAPK signaling cause side-effects for the normal growth of plants. Thus, plant MAPKs can reversely phosphorylate MAPKKKs to regulate the MAPK cascade, precisely controlling signal transduction or responses [8].

## 2. Component of Rice MAPK Cascades

The rice genome contains 75 OsMAPKKKs, 8 OsMAPKKs and 15 OsMAPKs [9,10]. The OsMAPKKKs, occupying the largest group of rice MAPK cascade proteins, are divided into three families, including 43 Raf family OsMAPKKKs, 22 MEKK family OsMAPKKKs, and 10 ZIK family OsMAPKKKs [10]. Although the rice genome harbors eight OsMAPKKs, two of them could not be detected on transcriptional levels in different rice tissues, and are considered as pseudogenes, thus there are only six functional MAPKKs in rice [11]. The MAPKs are divided into two subtypes, T-E-Y and T-D-Y, according to the conserved T-X-Y motif in their active loop that specifically phosphorylated by MAPKKs. Of these, T-E-Y subtype contains five OsMAPKs, T-D-Y subtype has 10 OsMAPKs [11].

So far, nine of 75 OsMAPKKKs (OsMAPKKK1, OsMAPKKK6, OsMAPKKK10, OsMAPKKK11, OsMAPKKK18, OsMAPKKK24, OsMAPKKK43, OsMAPKKK62, OsMAPKKK63), five of eight OsMAPKKs (OsMAPKK1, OsMAPKK3, OsMAPKK4, OsMAPKK6, OsMAPKK10-2), and ten of 15 OsMAPKs (OsMAPK3, OsMAPK4, OsMAPK6, OsMAPK7, OsMAPK14, OsMAPK16, OsMAPK17-1, OsMAPK17-2, OsMAPK20-4, OsMAPK20-5) have been functionally characterized (Table 1). OsMAPKKKs, OsMAPKKs and OsMAPKs play roles in rice growth and development, such as plant architecture, leaf morphology, embryogenesis, seed development, seed dormancy, panicle size, and grain size. They also have crucial roles in response to biotic stress, positively or negatively regulating rice resistance to pathogens including *Magnaporthe oryzae* (*M. oryzae*), *Xanthomonas oryzae* pv. *oryzae* (*Xoo*), *Xanthomonas oryzae* pv. *oryzicola* (*Xoc*), *Burkholderia glumae* (*B. glumae*) and *Rhizoctonia solani* (*R. solani*), and to herbivores including striped stem borer (SSB) and brown planthopper (BPH). Similarly, these MAPKs function in response to abiotic stress, such as drought, cold, salt or submergence stress. Alternatively, a number of MAPKs participate in phytohormone accumulation, signal transduction or response, such as abscisic acid (ABA), salicylic acid (SA), jasmonic acid (JA), ethylene (ET), brassinosteroids (BR) or cytokinin (CK).

A host of plant MEKK family MAPKKKs are considered *bona fide* MAPKKKs, which can directly phosphorylate the activation loop of downstream MAPKKs, and act the same pattern similar to MAPKKKs of animals and yeast. In contrast, several plant Raf family MAPKKKs can interact with downstream MAPKKs to promote MAPKKs degradation or suppress phosphorylation activity of MAPKKs on its direct downstream substrate MAPKs, but not directly phosphorylate and activate MAPKKs. Thus, some references suggest to exclude these plant Raf family MAPKKKs from *bona fide* MAPKKKs [54,55,56,57,58]. Compared with OsMAPKKs and OsMAPKs, rice has a great number of OsMAPKKKs, and more than half are Raf family MAPKKKs, the non-canonical MAPKKKs. Among the nine functionally characterized OsMAPKKKs, six of them (OsMAPKKK10, OsMAPKKK11, OsMAPKKK18, OsMAPKKK24, OsMAPKKK62, OsMAPKKK63) belong to MEKK family MAPKKKs, of which OsMAPKKK10, OsMAPKKK11, OsMAPKKK18, and OsMAPKKK24 have been validated as *bona fide* MAPKKKs with the capacity that to directly phosphorylate and activate downstream MAPKKs [17,18,19,20,21]. Three functionally characterized OsMAPKKKs (OsMAPKKK1, OsMAPKKK6, OsMAPKKK43) are members of Raf family. Whether they can associate with or phosphorylate the downstream OsMAPKKs to activate or suppress the OsMAPKKs is unclear so far [12,13,14,15,16,22,23]. Of the ten functionally studied OsMAPKs, OsMAPK3, OsMAPK4 and OsMAPK6 are T-E-Y subtype MAPKs, the other seven (OsMAPK7, OsMAPK14, OsMAPK16, OsMAPK17-1, OsMAPK17-2, OsMAPK20-4, OsMAPK20-5) are T-D-Y subtype MAPKs. Only a few of them are validated as substrates and can be phosphorylated by upstream OsMAPKKs, such as OsMAPK3, OsMAPK6, OsMAPK7, and OsMAPK14 [24,28,30,33].

A complete MAPK cascade consists of MAPKKK, MAPKK and MAPK [3]. Several integrative rice MAPK cascades have recently been identified involved in diverse physiological processes. OsMAPKKK11/18/24-OsMAPKK4/5-OsMAPK3/6 cascades function downstream of OsCERK1-OsRLCK185 complex and confer rice resistance to fungal pathogen *M. oryzae* [20,21]. OsMAPKKK10-OsMAPKK4-OsMAPK6 cascade plays critical roles in rice grain morphogenesis, rice panicle development, BR homeostasis and signaling pathway [17,18,19]. OsMAPKKK62-OsMAPKK3-OsMAPK7/14 cascades affect ABA signal transduction, ABA content and seed dormancy [24]. OsMAPKKK63-OsMAPKK1-OsMAPK4 cascade regulates salt stress response [25,26]. Apart from these complete MAPK cascades, the other cascades consisting of either OsMAPKKK-OsMAPKK or OsMAPKK-OsMAPK lack downstream substrate OsMAPKs or upstream OsMAPKKKs, respectively. OsMAPKKK63-OsMAPKK6 cascade regulates seed dormancy, while the downstream substrate OsMAPKs have not been identified [25]. Additionally, OsMAPKK3-OsMAPK7 cascade is involved in rice resistance to bacterial pathogen *Xoo* [30], and OsMAPKK6-OsMAPK3 cascade participates in cold stress response [34,35], but their corresponding upstream OsMAPKKKs are unclear.

## 3. Complicated Action Mode of Rice MAPK Cascades

Compared with 75 OsMAPKKKs and 15 OsMAPKs, rice contains six functional OsMAPKKs, implying that an OsMAPKK can be phosphorylated by multiple upstream OsMAPKKKs, and similarly a OsMAPKK can phosphorylate and activate several downstream OsMAPKs as its substrate. It seems that OsMAPKKs function as key nodes or hubs in MAPK cascades [59]. Of the five functionally deciphered OsMAPKKs, OsMAPKK4 typically acts as a hub of MAPK cascades, since OsMAPKKK10, OsMAPKKK11, OsMAPKKK18, and OsMAPKKK24 can separately phosphorylate OsMAPKK4 [20,21]. OsMAPKK4, in turn, can simultaneously phosphorylate and activate both OsMAPK3 and OsMAPK6 [33]. When phosphorylated by different OsMAPKKKs after rice sensing different external signals, OsMAPKK4 can select different downstream OsMAPKs for subsequential signal transduction. After rice sensing chitin-triggered signal or recognizing fungal pathogen *M. oryzae* invasion, OsMAPKK4 is rapidly phosphorylated by OsMAPKKK11, OsMAPKKK18 or OsMAPKKK24, then OsMAPKK4 subsequently phosphorylates OsMAPK3 and OsMAPK6 to transfer signals to downstream transcription factors, promoting rice resistance to *M. oryzae* [20,21]. However, when rice senses a BR signal, OsMAPKK4 is phosphorylated by OsMAPKKK10, then OsMAPKK4 phosphorylates OsMAPK6 for downstream signal transduction [17,18,19].

As mentioned above that different MAPKKKs can phosphorylate a MAPKK, whereas, different MAPKKs can also phosphorylate a MAPK. For example, OsMAPKK1, OsMAPKK3, OsMAPKK4, OsMAPKK5, and OsMAPKK10-2 can interact with and phosphorylate OsMAPK6, mediating rice resistance to fungal and bacterial pathogens or regulating rice growth and developmental responses [17,18,27,28,33]. OsMAPKK4, OsMAPKK6, and OsMAPKK10-2 can associate with and phosphorylate OsMAPK3, being involved in defense response, cold tolerance, and drought tolerance, respectively [28,29,33,34,54,60].

In turn, a MAPKK can phosphorylate and activate several MAPKs, including OsMAPKK1 can target OsMAPK4 and OsMAPK6 [26,54,60], OsMAPKK10-2 phosphorylates OsMAPK3 and OsMAPK6 [27,28,29], OsMAPKK3 interacts with OsMAPK6, OsMAPK7 and OsMAPK14 [24,30,54,60], OsMAPKK6 associates with OsMAPK3, OsMAPK4 and OsMAPK6 [29,54,60,61]. The present data uncover that a MAPKK can interact with several MAPKs to play roles in different physiological processes. OsMAPKK10-2 regulates rice resistance to fungal pathogen *M. oryzae* and bacterial pathogen *Xoc* by activating OsMAPK6, while modulates drought tolerance via activation of OsMAPK3 [27,28]. It indicates that there are complex action modes of rice MAPK cascades, which largely determine their multiple roles.

## 4. Controlling Growth and Development by Rice MAPK Cascades

Like MAPK cascade regulates cell proliferation and cell differentiation to influence plant growth or development, some members of rice MAPK cascades control embryogenesis, fertility, seed development, grain performance, panicle morphogenesis, and architecture (Table 1).

MAPK cascades play critical roles in rice embryogenesis. Functional analysis of loss-of-function mutants of *OsMAPK6* reveals that *OsMAPK6* affects the differentiation of L1 layer cells during early embryogenesis to arrest the embryonic development at the globular stage via influencing GA and auxin synthesis [45]. By screening of a series of osmapk mutants generated via CRISPR-Cas9 technology, heterozygous *osmapk6* mutants can produce homozygous *osmapk6* seeds but with abnormal embryo [42], while heterozygous *osmapk4* mutants do not produce homozygous *osmapk4* seeds, implying *OsMAPK6* and *OsMAPK4* influence seed development [42].

**Figure 1:**
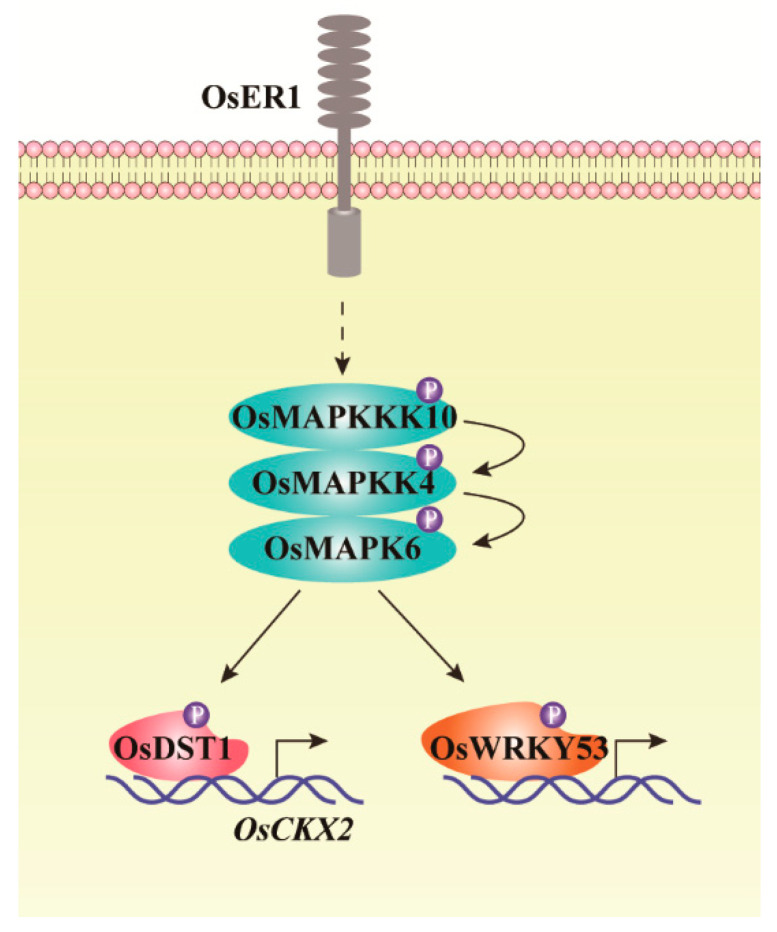
Schematic diagram of MAPK cascade regulating rice panicle and grain development. Plasma membrane localized receptor kinase OsER1 acts directly upstream of OsMAPKKK10-OsMAPKK4-OsMAPK6 cascade. The phosphorylated OsMAPK6 can phosphorylate OsDST1, then the phosphorylated OsDST1 targets and promotes the transcription of *OsCKX2*, regulating rice panicle morphology. Simultaneously, OsMAPKKK10-OsMAPKK4-OsMAPK6 cascade can phosphorylate OsWRKY53 to regulate rice BR signal transduction to alter rice architecture [17,18,19,63].

MAPK cascades play key roles in rice grain size and panicle morphogenesis. By screening mutants with altered grain size, *smg1* mutant with multiple phenotypes, including small grains, erect leaves, dense and erect panicles has been identified. Genetic analysis indicates that *smg1* is loss-of-function of *OsMAPKK4*, which influences cell proliferation and BR signal [32]. Meanwhile, a natural mutant, *dsg1* with pleiotropic phenotypes, including significant dwarfism, small grains, erect and dark-green leaves has been identified. Complement genetic assay indicates that pleiotropic phenotypes of *dsg1* are caused by loss of *OsMAPK6*. Subsequently, genetic analysis indicates that OsMAPKK4 acts upstream of OsMAPK6, by phosphorylating and activating OsMAPK6 to influence cell proliferation [62]. Recently, OsMAPKKK10 has been validated to regulate rice grain size and panicle development via activating OsMAPKK4-OsMAPK6 cascade by a series of genetic and biochemical analysis [17,18]. The OsMAPKKK10-OsMAPKK4-OsMAPK6 is so far the only completely known MAPK cascade, which regulates rice growth and development. The latest data have uncovered that plasma membrane localized receptor kinase OsER1 acts directly upstream of OsMAPKKK10-OsMAPKK4-OsMAPK6 cascade. The phosphorylated OsMAPK6 can subsequently phosphorylate OsDST1, then the phosphorylated OsDST1 binds to the promoter of *OsCKX2* and promotes the transcription of *OsCKX2* [17,18,19]. The whole signal transduction pathway, from plasma membrane OsER1 to cytoplasm OsMAPKKK10-OsMAPKK4-OsMAPK6, then to nucleus OsCKX2, uncovers a practically perfect genetic regulating network which regulates rice panicle morphogenesis, except the only gap between OsER1 and OsMAPKKK10 (Figure 1).

MAPK cascades function in rice architecture formation via modulating leaf morphology and plant height. The *osmapkkk43* mutant caused by a T-DNA insertion shows an increased leaf angle. Following cell biology and genetic assays indicate that *OsMAPKKK43* regulates mechanical tissue formation to modify leaf lamina joint by modulating secondary wall synthesis [22,23].

## 5. Coordinating Biotic Stress Response by Rice MAPK Cascades

A great number of plant MAPK cascades, especially of Arabidopsis MAPK cascades, have positive or negative effects on pathogens or insects invasion. Several rice MAPK cascades have been validated to coordinate biotic response and trigger resistance to bacterial and fungal pathogens or herbivores (Table 1).

**Figure 2:**
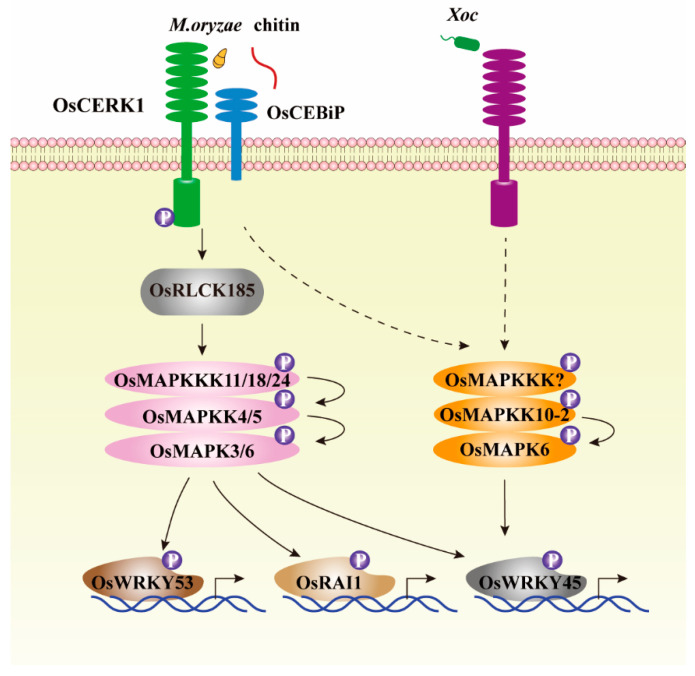
MAPK cascades are downstream of pattern recognition receptors complex and regulate rice immune response. After recognizing chitin or *M. oryzae*, rice pattern recognition receptors complex including OsCERK1 and OsCEBiP that localized at plasma membrane can phosphorylate OsRLCK185 to activate OsMAPKKK11/18/24-OsMAPKK4/5-OsMAPK3/6 cascades, leading to activation of numerous immune-related transcription factors, such as OsWRKY45, OsWRKY53, and OsRAI1 to initiate rice defense response [20,21,64,65]. Both fungal pathogen *M. oryzae* and bacterial pathogen *Xoc* can activate OsMAPKK10-2-OsMAPK6 cascade via unknown OsMAPKKK to enhance biochemical activity of OsWRKY45, thereby triggering immune response to pathogens [27,28,29].

MAPK cascades confer resistance to fungal pathogens. At least two OsMAPKKKs, two OsMAPKKs, and four OsMAPKs have been reported to be involved in resistance to fungal pathogen *M. oryzae*. Both OsMAPKKK1 and OsMAPKKK24 play positive roles in resistance to *M. oryzae*, while employing different molecular mechanisms. *OsMAPKKK1* triggers resistance to *M. oryzae* by modulating ET biosynthesis to inhibit fungi penetration into rice cells, and *OsMAPKKK24* by activating OsMAPKK4-OsMAPK6 cascade [12,13,21]. Both *OsMAPKKK11* and *OsMAPKKK18* are activated by chitin, the fungal microbial-associated molecular pattern. However, there is no direct evidence to confirm these two genes enhancing rice resistance to *M. oryzae* [20]. Of the four OsMAPKs to be involved in resistance to fungal pathogen, *OsMAPK3* and *OsMAPK16* negatively regulate resistance to *M. oryzae* [36,37,48], while *OsMAPK20-5* positively confers resistance to *M. oryzae* [53]. *OsMAPK6* is transcriptionally induced by sphingolipid elicitor and chitin, implying that *OsMAPK6* possibly plays role in rice-*M. oryzae* interactions [33,34]. OsMAPKK10-2 can phosphorylate OsMAPK6, causing activated OsMAPK6 to subsequently phosphorylate and enhance the biochemical activity of downstream transcription factor OsWRKY45 to trigger rice resistance to *M. oryzae* [27]. Similarly, OsMAPKK4 phosphorylates and activates OsMAPK3 and OsMAPK6 to confer resistance to *M. oryzae*, through accumulation of diterpenoid phytoalexin, momilactones and phytocassanes [33]. However, the underlying mechanisms, why phosphorylated OsMAPK3 and OsMAPK6 by different upstream OsMAPKKs, cause susceptibility and confer resistance to *M. oryzae*, respectively, are unclear. Apart from being involved in resistance to fungal pathogen *M. oryzae*, *OsMAPK20-5* has been reported simultaneously to be involved in resistance to fungal pathogen *R. solani* [53]. By integrating the characterized MAPK cascades, OsMAPKKK11/18/24-OsMAPKK4/5-OsMAPK3/6 cascades are the complete MAPK cascades, which mediate *M. oryzae*-triggered signal transduction and promote rice resistance to *M. oryzae* (Figure 2).

MAPK cascades trigger resistance to bacterial pathogens. Up to now, one OsMAPKKK, two OsMAPKKs, and six OsMAPKs have been referenced to be involved in resistance to bacterial pathogens, *Xoo*, *Xoc* or *B. glumae*. *OsMAPKKK1* negatively regulates resistance to *Xoo* by modulating accumulation of JA and SA [13]. OsMAPKK10-2 functions as a positive regulator in response to *Xoc* by activating downstream OsMAPK6 [28]. OsMAPKK3 also functions as a positive regulator but in response to *Xoo* by activating downstream OsMAPK7, with the signal transduction that the activated OsMAPK7 phosphorylates and activates the transcription factor OsWRKY30 to enhance rice resistance to *Xoo* [30]. Of the six OsMAPKs conferring resistance to bacterial pathogen, *OsMAPK3* and *OsMAPK16* play negative roles in response to *Xoo* [37,48], while *OsMAPK7* and *OsMAPK17-1* play positive roles in resistance to *Xoo* [30,49]. Interestingly, *OsMAPK4* positively confers resistance to *Xoo* by promoting the accumulation of JA and SA, while it negatively influences resistance to *Xoo* by negatively regulating systemic acquired resistance, because both *OsMAPK4* overexpressing plants and *osmapk4* mutant exhibit enhanced resistance to *Xoo* [40,41]. In addition, *OsMAPK3* is also involved in resistance to *B. glumae*, a soil bacterium [36].

MAPK cascades also have roles in resistance to herbivores. Although a number of rice MAPK genes show diverse transcriptional patterns upon herbivores BPH and SSB infection, only one OsMAPKK and three OsMAPKs have been validated exhibiting resistance to BPH or SSB. OsMAPKK3 functions as a positive regulator in rice-BPH interactions by modulating herbivory-induced phytohormone dynamics [31]. In line with OsMAPKK3, OsMAPK3 and OsMAPK4 also act as positive regulators conferring resistance to SSB with partly similar mechanisms. *OsMAPK3* triggers resistance to SSB by regulating JA signaling pathway and promoting accumulation of herbivory-induced trypsin protease inhibitors [39], and *OsMAPK4* confers resistance to SSB by regulating JA, ET and SA signaling pathways [43]. Additionally, *OsMAPK20-5* which transcriptionally induced by gravid female BPH, negatively regulates rice resistance to BPH via suppressing the accumulation of ET and NO [52]. It seems that these three OsMAPKs largely regulate resistance to herbivores by modulating phytohormone signaling pathway.

## 6. Conferring Resistance to Abiotic Stress by Rice MAPK Cascades

**Figure 3:**
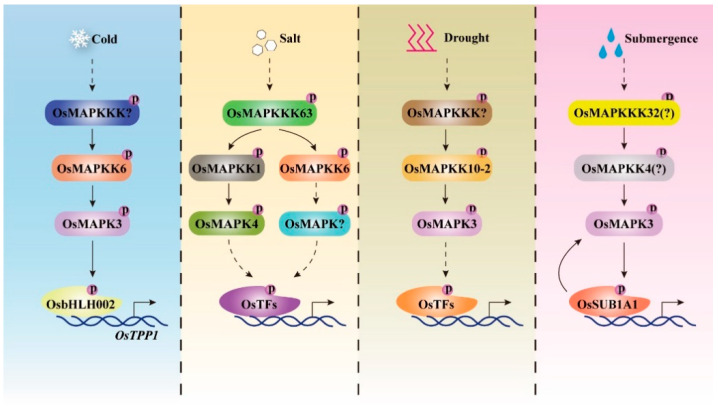
Rice MAPK cascades are activated by different abiotic stress signals and confer tolerance to diverse abiotic stress in rice. A variety of abiotic stress, such as cold, salt, drought, and submergence could activate diverse rice MAPK cascades, which play critical roles in triggering rice resistance to these stresses [16,25,26,27,28,34,35,36,67,68]. OsMAPKKK63-OsMAPKK1-OsMAPK4 is the only known cascade conferring salt tolerance, while its downstream substrates have not been identified.

In addition to biotic stress, rice MAPK cascades have also been confirmed conferring abiotic stress responses, under such as salt, drought, cold, or submergence. For example, OsMAPK3 is the fully characterized MAPK cascade protein which kinase activity is induced by a series of abiotic stress including drought, salt, cold and submergence. The *OsMAPK3* overexpressing plants show enhanced resistance to these different abiotic stress [36]. The following research indicates that OsMAPKK6 which acts upstream of OsMAPK3 enhances rice cold tolerance [34,35]. The mechanism of OsMAPKK6-OsMAPK3 cascade being involved in cold tolerance is recently been deciphered, with that the activated OsMAPK3 interacts with and phosphorylates OsbHLH002/OsICE1, in turn phosphorylated OsbHLH002/OsICE1 binds and promotes the expression of *OsTPP1* to cause trehalose accumulation, thereby increasing cold tolerance for rice plants [66]. Whereas, OsMAPK3 has roles in drought tolerance by acting as substrate for OsMAPKKK10-2, the underlying molecular mechanism is unclear [28]. Furthermore, OsMAPK3 has positive effect on salt tolerance by attenuating the reactive oxygen species accumulation [67]. These results demonstrate that OsMAPK3 confers tolerance to salt, drought, or cold stress probably by phosphorylating different substrates (Figure 3).

For other MAPKs, OsMAPKK1, its kinase activity is induced by salinity, plays positive roles towards salt stress by phosphorylating and activating downstream substrate OsMAPK4 [26]. Recently, OsMAPKKK63 is found to associate with OsMAPKK1 to enhance rice resistance to salt stress [25]. Thus, a complete MAPK cascade consisting of OsMAPKKK63-OsMAPKK1-OsMAPK4 is identified, which positively promotes rice for salinity tolerance (Figure 3). OsMAPKKK6 functions as a positive regulator towards drought stress by regulating reactive oxygen species scavenging, while its downstream OsMAPKK or OsMAPK are unidentified till now [16].

## 7. Conducting Phytohormone Signal Transduction by Rice MAPK Cascades

**Figure 4:**
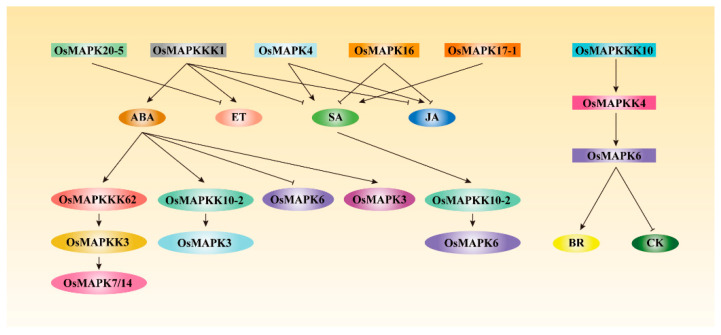
Rice MAPK cascades regulate or are involved in phytohormone accumulation, signal transduction or response. Rice MAPK cascades positively or negatively regulate phytohormone accumulation and signal transduction, or been directly or indirectly modulated by phytohormones, such as SA, JA, ABA, ET, BR, and CK [12,13,14,15,27,28,40,43,48,49]. The established OsMAPKKK62-OsMAPKK3-OsMAPK7/14 cascades are regulated by ABA, while the OsMAPKKK10-OsMAPKK4-OsMAPK6 cascade can regulate BR and CK signal transduction [17,18,19,24].

As key molecules linking extracellular and intracellular signal transduction, MAPK cascades have been widely reported to participate in phytohormone accumulation, signaling pathways or response, such as ABA, SA, JA, CK, BR or ET. MAPK cascade-mediated phytohormone signal transduction largely contributes to their diverse roles in growth and developmental responses, or biotic and abiotic stress responses. For example, SA treatment can upregulate the transcription of *OsMAPKK10-2*, the activated OsMAPKK10-2 phosphorylates and enhances the activity of OsMAPK6, triggering the SA signaling pathway to improve rice resistance to bacterial pathogen *Xoc* and fungal pathogen *M. oryzae*. Reversely, ABA treatment can induce the transcription of both *OsPTP1* and *OsPTP2*, encoding two tyrosine phosphatases, which two can dephosphorylate the tyrosine residues at the T-E-Y motif of OsMAPK6 and cause the inactivation of OsMAPK6, resulting in decreased resistance to fungal pathogen *M. oryzae* and increased sensitivity to drought stress [27,28]. Lately, OsMAPK6 is reported to be a substrate of OsMAPKKK10-OsMAPKK4 cascade being involved in BR signal transduction, modulating rice architecture and grain size [17,18,19]. Furthermore, activated OsMAPK6 by OsMAPKKK10-OsMAPKK4 cascade can also regulate CK metabolism to alter rice panicle development. OsMAPK6 interacts with and phosphorylates OsDST1, then the phosphorylated OsDST1 binds and promotes the transcription of *OsCKX2*, which encodes the cytokinin oxidase/dehydrogenase. Thereby, activated OsCKX2 accelerates catalyzing the degradation of active CK to alter the number of rice spikelets [19]. Thus, the OsMAPKKK10-OsMAPKK4-OsMAPK6 cascade is closely associated with CK homeostasis in determining rice panicle development (Figure 4). The series of results suggest that different phytohormone signaling pathways can modulate OsMAPK6 function in diverse physiological processes, and OsMAPK6 could also phosphorylate different downstream substrates to regulate phytohormone homeostasis, fine-tuning rice growth and developmental responses as well as biotic and abiotic stress responses.

In the same way, other MAPK cascades are involved in phytohormone response. For example, the OsMAPKKK62-OsMAPKK3-OsMAPK7/14 and OsMAPKK10-2-OsMAPK3 cascades are associated with ABA signal transduction, regulating rice seed dormancy [24,28]. OsMAPKKK1 acts as a positive regulator in ABA and ET signaling pathways, while as a negative regulator in JA and SA signaling pathways [12,13,14,15], implying that OsMAPKKK1 probably interacts with different proteins or phosphorylates different downstream OsMAPKKs to play roles in diverse phytohormone signaling pathways. Similarly, OsMAPK4 positively regulates the accumulation of JA and SA, and OsMAPK17-1 positively regulates the accumulation of SA, while OsMAPK16 negatively regulates the accumulation of JA and SA, and OsMAPK20-5 negatively affects the synthesis of ET [40,43,48,49]. These data demonstrate that rice MAPK cascades regulate or involve in complex phytohormone accumulation, signaling pathways or response (Figure 4).

## 8. Complex Substrates of Rice MAPK Cascades

MAPK cascades play roles relying on phosphorylating a variety of downstream substrates, which include transcription factors, chromatin remodeling factors, kinases or other enzymes, and other proteins. So far, nine substrates for OsMAPK3, six substrates for OsMAPK6, one substrate for both OsMAPK4 and OsMAPK7, two substrates for both OsMAPK14 and OsMAPK17-1 have been identified and functionally characterized. Of which majority of substrates are composed of transcription factors (TF), such as WRKY or bHLH, few of them are kinase or other proteins (Table 2). For example, OsMAPK3 phosphorylates OsCDPK18 and OsRAI1 to confer rice resistance to fungal pathogen *M. oryzae* [38,64], acts on OsWRKY30 to confer resistance to bacterial pathogen *Xoo* [69], while phosphorylates OsbHLH002, OsZFP213, SUB1A1 and OsWRKY30 to alter stress tolerance, such as cold, salt, submergence and drought, respectively [66,67,68,69]. Occasionally, an OsMAPK could phosphorylate different substrates to regulate the same signaling pathway or play the same roles.

OsMAPK3 phosphorylates kinase OsCDPK18 and TF OsRAI1, totally improving rice resistance to *M. oryzae* [38,64]. In the same way, OsMAPK6 acts on three different TFs, OsWRKY53, OsWRKY45, and OsRAI1, to collectively trigger rice resistance to *M. oryzae* [29,64,65]. Reversely, different OsMAPKs target the same substrate participating in the same physiological processes. For example, both OsMAPK3 and OsMAPK6 phosphorylate OsRAI1 to positively confer rice resistance to fungal pathogen *M. oryzae* [64], and phosphorylate OsWRKY70 to enhance rice resistance to herbivores, BPH and SSB [69,73]. OsMAPK3, OsMAPK7, and OsMAPK14 all can phosphorylate OsWRKY30 to promote rice resistance to bacterial pathogen *Xoo* and modulate rice drought tolerance [69,73]. These data suggest the complex relationships between OsMAPKs and their diverse substrates.

## 9. Conclusions

Tremendous progress has been made to decipher the multiple functions of rice MAPK cascades, with several complete MAPK cascades have been uncovered, including OsMAPKKK11/18/24-OsMAPKK4/5-OsMAPK3/6 cascades, OsMAPKKK10-OsMAPKK4-OsMAPK6 cascade, OsMAPKKK63-OsMAPKK1-OsMAPK4 cascade, and OsMAPKKK62-OsMAPKK3-OsMAPK7/14 cascades. However, due to over 98 MAPK genes in rice, a large number of them have not been functionally characterized. The gaps need to be filled, including which proteins target OsMAPKKKs, which OsMAPKKs can be phosphorylated by OsMAPKKKs, which OsMAPKs can be phosphorylated by OsMAPKKs, and which proteins can be subsequently phosphorylated by OsMAPKs. Previously, yeast two hybrid and in vitro phosphorylation assay are the main methods for MAPK substrates identification, while these two methods may produce false negatives and positives [1,60]. Thus, quantitative phosphoproteomic and immunoprecipitation-mass spectrometry methods have recently been used to identify protein kinase substrates and study the function of protein kinases [78,79]. Therefore, the combination of quantitative phosphoproteomic, immunoprecipitation-mass spectrometry, in vitro phosphorylation, and genetic assays would be alternative strategies to uncover the function of MAPKs and identify their substrates. Furthermore, the same MAPK cascade can sense and mediate different signal transduction, playing roles in diverse physiological processes. However, the underlying mechanisms that a MAPK cascade precisely activates and phosphorylates different downstream substrates after sensing different upstream signals are still unclear. Resolving these putative concerns would fully accelerate to elucidate the underlying molecular mechanisms and molecular functions of rice MAPK cascades.

## Figures and Tables

**Table 1:** Function characterized rice MAPK genes.

Gene Name ^a^	Gene Locus ^b^	Alternative Names	Biological Functions ^c^	References
*OsMAPKKK1*	Os03g06410	*SPL3/OsEDR1/* *OsACDR1*	*M. oryzae*^+^, *Xoo*^−^, SA^−^/JA^−^/ET^+^ accumulation, ABA^+^ response	[12,13,14,15]
*OsMAPKKK6*	Os02g50970	*OsDSM1*	Drought stress^+^	[16]
*OsMAPKKK10*	Os04g47240	*SMG2*	Panicle and grain size^+^, architecture^+^, BR^+^ response, CK^−^ accumulation	[17,18,19]
*OsMAPKKK11*	Os07g02780		Chitin response^+^	[20]
*OsMAPKKK18*	Os03g55560		Chitin response^+^	[20]
*OsMAPKKK24*	Os04g56530	*OsMAPKKKε*	*M. oryzae* ^+^	[21]
*OsMAPKKK43*	Os06g50920	*OsILA1*	Leaf morphology^−^	[22,23]
*OsMAPKKK62*	Os01g50420		Seed dormancy^−^, ABA^−^ response	[24]
*OsMAPKKK63*	Os01g50370		Salt stress^−^, seed dormancy^−^, ABA^−^ response	[25]
*OsMAPKK1*	Os06g05520	*OsMEK2*	Salt stress^+^	[26]
*OsMAPKK10-2*	Os03g12390	*OsMEK3*	*M. oryzae*^+^, *Xoc*^+^, drought stress^+^, SA^+^/ABA^+^ response	[27,28,29]
*OsMAPKK3*	Os06g27890	*OsMEK8a*	*Xoo*^+^, BPH^+^, seed dormancy^−^, ABA^−^ response	[24,30,31]
*OsMAPKK4*	Os02g54600	*SMG1*/*OsMEK6*	*M. oryzae*^+^, panicle and grain size^+^, architecture^+^, BR^+^ response, CK^−^ accumulation	[32,33]
*OsMAPKK6*	Os01g32660	*OsMEK1*	Cold and salt stress^+^	[34,35]
*OsMAPK3*	Os03g17700	*OsBIMK1/OsMAP1/OsMSRMK2/OsMPK5/OsMAPK2*/*OsMPK3*	*M. oryzae*^−^, *Xoo*^−^, SSB^+^, *B. glumae*^−^, cold and drought stress^+^, ABA^+^/JA^+^ response	[36,37,38,39]
*OsMAPK4*	Os10g38950	*OsMPK6*	*Xoo*^+-^, SSB^+^, salt stress^+^, seed development^+^, SA^+^/JA^+^ accumulation	[40,41,42,43]
*OsMAPK6*	Os06g06090	*OsMPK1/* *OsSIPK/DSG1*/ *OsMPK6*	*M. oryzae*^+^, *Xoc*^+^, embryogenesis, panicle and grain size^+^, SA^+^/BR^+^ response, ABA^−^/CK^−^ accumulation	[28,33,44,45]
*OsMAPK7*	Os06g48590	*OsMPK4/OsAMPK4/OsMPK7/* *OsMSRMK3*	*Xoo*^+^, seed dormancy^−^, ABA^−^ response	[24,30,46]
*OsMAPK14*	Os02g05480	*OsMAPK33/OsMPK3*/*OsMAPK3*	Seed dormancy^−^, ABA^−^ response	[24,47]
*OsMAPK16*	Os11g17080	*OsMPK15*	*M. oryzae*^−^, *Xoo*^−^, SA^−^/JA^−^ accumulation	[48]
*OsMAPK17-1* *OsMAPK17-2*	Os06g49430 Os02g04230	*OsMPK12/* *OsBWMK1* *OsBIMK2*/ *OsMPK13*	*Xoo*^+^, SA^+^ accumulation Transcriptionally induced by SA	[37,49] [50]
*OsMAPK20-4* *OsMAPK20-5*	Os01g47530 Os05g49140	*OsMPK8*/ *OsMPKG1* *OsMPK7*	Transcriptionally induced by ABA *M. oryzae*^+^, *R. solani*^−^, BPH^−^, ET^−^ accumulation	[51] [52,53]

^a^ The names of rice MAPKs are used according to reference [9,11,54]. ^b^ Locus ID from Rice Genome Annotation Release 7. ^c^ *M. oryzae*: *Magnaporthe oryzae*; *Xoo*: *Xanthomonas oryzae* pv. *oryzae*; *Xoc*: *Xanthomonas oryzae* pv. *oryzicola*; *B. glumae*: *Burkholderia glumae*; *R. solani*: *Rhizoctonia solani*; SSB: striped stem borer; BPH: brown planthopper; ABA: abscisic acid; SA: salicylic acid; JA: jasmonic acid; ET: ethylene; BR: brassinosteroids; CK: cytokinin. ^+^ Playing positive role. ^−^ Playing negative role.

**Table 2:** Substrates of rice OsMAPKs.

OsMAPK	Substrate	Substrate Protein	Evidence ^a^	Substrate Function	References
OsMAPK3	OsCDPK18	kinase	1,2	*M. oryzae* ^−^	[38]
OsMAPK3	OsbHLH002/ OsICE1	TF	1,2,3,4	Cold stress^+^	[66]
OsMAPK3	OsZFP213	TF	1	Salt stress^+^	[67]
OsMAPK3	OsDRB1	double-strand RNA binding protein	1,2,3	miRNA biogenesis	[70]
OsMAPK3	SUB1A1	TF	1,2,3,4	Submergence tolerance^+^	[68]
OsMAPK3	Bphi008a	Wir1-like protein	1	BPH^+^	[71,72]
OsMAPK3	OsRAI1	TF	1,2	*M. oryzae* ^+^	[64]
OsMAPK3	OsWRKY70	TF	1,2	BPH^−^, SA^−^/GA^−^ accumulation, SSB^+^, JA^+^/ET^+^ accumulation	[73]
OsMAPK3	OsWRKY30	TF	1,2,4	Drought stress^+^	[69]
OsMAPK4	OsWRKY45	TF	2	SA^+^ signaling	[29]
OsMAPK6	OsWRKY53	TF	1,2,4	*M. oryzae*^+^, grain size^+^, BR^+^ response	[63,65,74]
OsMAPK6	OsDST1	TF	1,2	Panicle and grain size^−^, CK^−^ accumulation	[19]
OsMAPK6	OsVQ13	VQ-motif containing protein	1	*Xoo*^+^, JA^+^ response	[75]
OsMAPK6	OsWRKY45	TF	2,4	*M. oryzae*^+^, SA^+^ signaling	[27,29]
OsMAPK6	OsRAI1	TF	1,2	*M. oryzae* ^+^	[64]
OsMAPK6	OsWRKY70	TF	1,2	SSB^+^, JA^+^/ET^+^ accumulation, BPH^−^, SA^−^/GA^−^ accumulation	[73]
OsMAPK7	OsWRKY30	TF	1,2,4	*Xoo*^+^, drought stress^+^	[30,69]
OsMAPK14	OsWRKY30	TF	1,2,4	Drought stress^+^	[69]
OsMAPK14	OsbHLH65	TF	1,2	Transcriptionally induced by *M. oryzae*, BPH, JA/SA treatment	[47]
OsMAPK17-1	OsWRKY33	TF	1,2	SA^+^ signaling	[76]
OsMAPK17-1	OsEREBP1	TF	1,2	Defense response^+^	[77]

^a^ Evidences provided to validate physiological substrates for the corresponding OsMAPK. 1, in vitro and in vivo interaction analysis. 2, in vitro phosphorylation analysis. 3, in vivo phosphorylation analysis. 4, mutation of phosphorylated S/T residues-based genetic analysis. ^+^ Playing positive role. ^−^ Playing negative role.

## Data Availability

Not applicable.
